# A network view of microRNA and gene interactions in different pathological stages of colon cancer

**DOI:** 10.1186/s12920-019-0597-1

**Published:** 2019-12-30

**Authors:** Jia Wen, Benika Hall, Xinghua Shi

**Affiliations:** Department of Bioinformatics and Genomics, College of Computing and Informatics, University of North Carolina at Charlotte, 9201 University City Blvd, Charlotte, 28223 NC USA

## Abstract

**Background:**

Colon cancer is one of the common cancers in human. Although the number of annual cases has decreased drastically, prognostic screening and translational methods can be improved. Hence, it is critical to understand the molecular mechanisms of disease progression and prognosis.

**Results:**

In this study, we develop a new strategy for integrating microRNA and gene expression profiles together with clinical information toward understanding the regulation of colon cancer. Particularly, we use this approach to identify microRNA and gene expression networks that are specific to certain pathological stages. To demonstrate the application of our method, we apply this approach to identify microRNA and gene interactions that are specific to pathological stages of colon cancer in The Cancer Genome Atlas (TCGA) datasets.

**Conclusions:**

Our results show that there are significant differences in network connections between miRNAs and genes in different pathological stages of colon cancer. These findings point to a hypothesis that these networks signify different roles of microRNA and gene regulation in the pathogenesis and tumorigenesis of colon cancer.

## Background

Colon cancer, which is reported to be one of the few curable cancers, is one of the most common cancers around the world. The complex progress of colon cancer stage induces the poor prediction prognosis of colon cancer. According to previous studies, there is a 92% 5-year relative survival rate [1, 2] in stage I colon cancer. For patients with stage II colon cancer, there are two stage subtypes: stage IIA and stage IIB colon cancers [1, 2]. There is an 87% 5-year relative survival rate for stage IIA and 63% for stage IIB. Similarly, for stage III colon cancer there are three subtypes: stage IIIA, IIIB and IIIC colon cancers [1, 2]. In patients with stage IIIA, the 5-year relative survival rate is 89%, for stage IIIB it is 69% and 53% for stage IIIC [1, 2]. When the cancer has reached stage IV and metastasized to other parts of the body, the 5-year relative survival rate is significantly decreased to approximately 12%. The drastic decrease in survival rate in colon cancer speaks to the need for better early diagnostic and prognostic procedures.

The role of pathologic prognostic markers is important in the advancement of personalized medicine and can help reduce the risk of recurrence, especially in high-risk patients with stage II colon cancer [3–5]. Due to the benefits of personalized medicine, these patients have an increased overall survival with therapies such as adjuvant chemotherapy. Gene expression signatures have shown much promise as prognostic markers [6]. For example, the progression of colon cancer is directly linked to the functional epithelial-mesenchymal transition (EMT) gene expression signatures [7]. Genes *ZEB*1 and *ZEB*2 are known repressors that regulate targets in the EMT pathway by changing the phenotype of normal cells to cancerous cells [8]. These genes are also known to be present in the beginning of metastasis.

Cell invasion and migration are also critical components in colon cancer progression. For instance, genes *PRKCQ* and *PRKCZ* are members of the protein kinase family and *PRKCZ* is often involved in cell survival and cell migration in different cancers such as ovarian cancer [9]. It has also been reported that *ARID*4*B* is a key player in pathogenesis and is classified as a metastasis modifier gene. Over-expression of this gene is thought to enhance the cell migration process as well as cell invasion. In contrast, the knockdown of *ARID*4*B*, causes metastasis of cancer cells to other regions of the body [10, 11].

More recently, microRNA (miRNA) expression profiles have been utilized as predictive markers for survival of colon cancer [12]. Previous studies have reported several miRNAs were relevant with poor survival and therapeutic outcome in colon cancer [13–15]. For example, *miR*−148, *miR*−26*a*−2 and *miR*−130*a* were identified to be significantly associated with a poor clinical prognosis [16]. Exploiting the downstream neighborhoods of genes with such a critical role in the pathogenesis of colon cancer provide long-term benefits in personalized medicine and adjuvant therapies. Studies have also found that the genetic changes varied among different stages of colon cancer, specially between stage II and stage III [3, 17, 18]. However, the genetic mechanism underlying these genetic changes that drive pathological stage progression are remain poorly studied.

There are some recent studies on delineating miRNAs and genes differentially expressed in different stages of colon cancer [19, 20]. These work started with conducting differentially expressed miRNAs and/or differentially expressed gene analysis to find the pathways that target genes and/or miRNAs are involved in different pathological stages of colon cancer. In our study, we intend to integrate miRNA and gene expression interactions from mining miRNA and gene expression profiles of cancer patients to investigate different network communities and patterns specific to different pathological stages of colon cancer. We hypothesized that as colon cancer progresses, there are unique functional patterns present in early stages that are not present in later stages and vice versa. We believe that identifying these functional signatures in different pathological stages can lead to improved prognosis and better understanding in the stage progression of colon cancer. Ultimately, the identification and analysis of the evolution and dynamics of these miRNA-gene networks will improve our understanding of the etiology and treatment of colon cancer. Using The Cancer Genome Atlas (TCGA) project [21] colon cancer data [22], we developed a novel strategy to integrate miRNA and gene expression data, incorporate known miRNA and gene targets, and protein protein interactions to generate a more comprehensive view of the miRNA-gene interactions.

## Methods

**Fig. 1:**
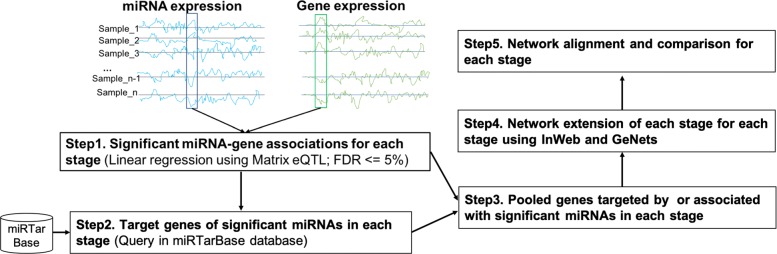
An illustration of our analysis workflow

Our integrative strategy for this work can be summarized as a workflow containing the following five steps (Fig. 1) after preprocessing the data.

**Step 1.** For each pathological stage, we conducted an association analysis between miRNA expression and gene expression quantifications in the same patients respectively using MatrixeQTL [23]. Those miRNA and gene associations that are statistically significant are kept for further analysis.

**Step 2.** For the miRNAs that are significantly associated with gene expression variations identified in **Step 1**, we obtained their target genes by retrieving from miRNA target gene databases like miRTarBase[24].

**Step 3.** We generated a gene set directly associated with or targeted by miRNAs for each pathological stage by merging all genes significantly associated with miRNAs output from **Step 1** and all of the targeted genes of those miRNAs significantly associated with gene expression obtained from **Step 2**. We then conducted the Gene Ontology (GO) Enrichment analysis for the gene set of each pathological stage to compare the functional differences among four stages.

**Step 4.** We overlapped the gene set directly associated with or targeted by miRNAs to InWeb, a comprehensive interaction database, to generate a preliminary miRNA-gene network in each pathological stage. We then expanded these preliminary miRNA-gene networks by adding interacting genes using two different methods and generated an integrated miRNA-gene network for each stage. The integrated miRNA-gene network for each pathological stage is now composed of miRNAs significantly associated with gene expression changes, the genes associated with or targeted by these miRNAs, and extended genes interacting with these genes.

**Step 5.** Finally, we performed community identification, network alignment, and statistical test to investigate the differences among the integrated miRNA-gene networks of all of the four pathological stages.

### Data preprocessing

The data in this study was first downloaded from TCGA (download date: June 11, 2015). Specifically, we retrieved miRNA expression data, gene expression data and clinical profiles for all colon cancer patients. The miRNA and gene expression data, quantified from miRNA and RNA sequencing respectively, were then preprocessed by first removing samples with missing data. We retained all clinical profiles of patients whose pathologic stage was known. If the sample stage was unknown, we did not include that sample in the study. We hence collected all samples with pathological stages marked as pathological stage I, II, III, and IV. We then conducted the analysis on these samples in each pathological stage respectively. We filtered the miRNA and gene expression matrices by removing those with over 10% of missing values in the samples. To reduce the variation between samples, we utilized inverse quantile normalization to normalize the miRNA and gene expression data separately. The quantile normalized miRNA and gene expression matrices are then used for this study.

### Identification of associations between miRNA and gene expression

We performed miRNA-gene association analysis between the miRNA expression and gene expression profiles in four pathological stages separately. We identified significant associations between miRNAs and genes where they are on the same chromosome and located across different chromosomes. The reference genome we used was GRCH38 with the gene reference of gencode.v29.annotation.gff3 downloaded from [25], and the miRNA reference of hsa.gff3 was downloaded from [26–30] The association analysis was performed using Matrix eQTL [31], which is an R package that uses matrix operations to identify pairwise associations. We conducted miRNA-gene association analysis by running a linear regression model using Matrix eQTL for each pair of miRNA and gene, and selected significant miRNA-gene associations using a 5% cutoff of false discovery rate (FDR). For each pathological stage, we extracted all of the experimentally validated target genes of those miRNAs asscociated with gene expressions from the miRTarBase target database [24]. At this point, we generated a target and associated gene set of miRNAs for each pathological stage by merging the genes associated with miRNAs and the genes targeted by miRNAs for further analysis.

### Gene Ontology analysis of miRNA associated and targeted genes

We conducted GO enrichment analysis for target and associated gene set of miRNAs (GO annotation released 2018-12-01) for each stage using the PANTHER over-representation test (Fisher’s exact test with a Bonferroni-corrected p-value cutoff at 0.05) [32, 33]. Using the 20,996 human whole-genome genes as background, we performed GO enrichment analysis of the genes for three categories including molecular function, biological process and cellular component.

### The construction of integrated miRNA-gene networks

To explore the genetic network induced by miRNA expression, we constructed a miRNA-gene interaction network for each pathological stage using the following strategy. First, we overlapped the gene set, composed of miRNA associated genes and targeted genes, with the genes in an interaction database InWeb [34, 35]. InWeb is a comprehensive protein-protein interaction network that integrated various interaction resources and was constructed using a stringent orthology majority-voting scheme [35]. We used InWeb as a template network for our network extension since it is dense and covers a wide spectrum of interaction information among genes. This overlapping process generated a preliminary miRNA-gene network for each pathological stage that is composed of miRNAs, their associated and targeted genes, and direct interactions among these genes.

Next, we expanded our miRNA and gene expression network for each stage using two methods. The first method is called GeNets [36], which uses a randomization approach to choose a new node to connect, based on a known network structure from InWeb [34, 35]. The second method we applied was a spin-glass model [37–39] that uses simulated annealing to identify the communities in a network. Our network extension process thus includes two stages. First, we used a scalable spin-glass algorithm in R’s igraph library [40] to identify all communities in the preliminary network including InWeb interactions among the associated and targeted genes of miRNAs. Second, we selected the most connected genes in the communities to find their downstream genes. The final integrated miRNA-gene network for each pathological stage was then constructed by including the miRNAs, the associated and targeted genes of these miRNAs, and the communities and extended neighborhood genes of the these associated and targeted genes. With this strategy, we constructed the final miRNA-gene network for each pathological stage that captures the direct and indirect effect of miRNAs on gene expression and provides a network view of how miRNAs affect gene expression in different pathological stages.

### Network analysis and alignment

We conducted comparative network analysis and network alignment among the integrated miRNA-gene networks across different pathological stages of colon cancer to investigate the differences in network properties and topology that may indicate disease progression. We first calculated important network properties of the four miRNA-gene networks for the four pathological stages. Specifically, we calculated the following four network properties, namely edge similarity, centrality, diameter and betweenness. Edge similarity indicates the similarity of edges/connections in two networks. Here we calculated the edge similarity as the proportion of common edges between stage I and all other stages respectively. Network diameter measures the length of the longest path from one vertex to another in a network and indicates the size of a network. Network centrality indicates the importance of vertices within a network. The betweenness of a network denotes the degree to which nodes stand between each other and is an important measurement of network centrality. We also calculated the degree distribution of all nodes in each network. We then performed network alignment to investigate how these miRNA-gene networks in different pathological stages align and differ in network topology. We utilized the “GraphAlignment” package in R [41] that aligns two networks or graphs using the adjacency matrices of two networks. We can thus identify subnetworks specific to each stage based on common nodes in the intersection of two networks.

## Results

### Significant miRNA-gene associations

**Fig. 2:**
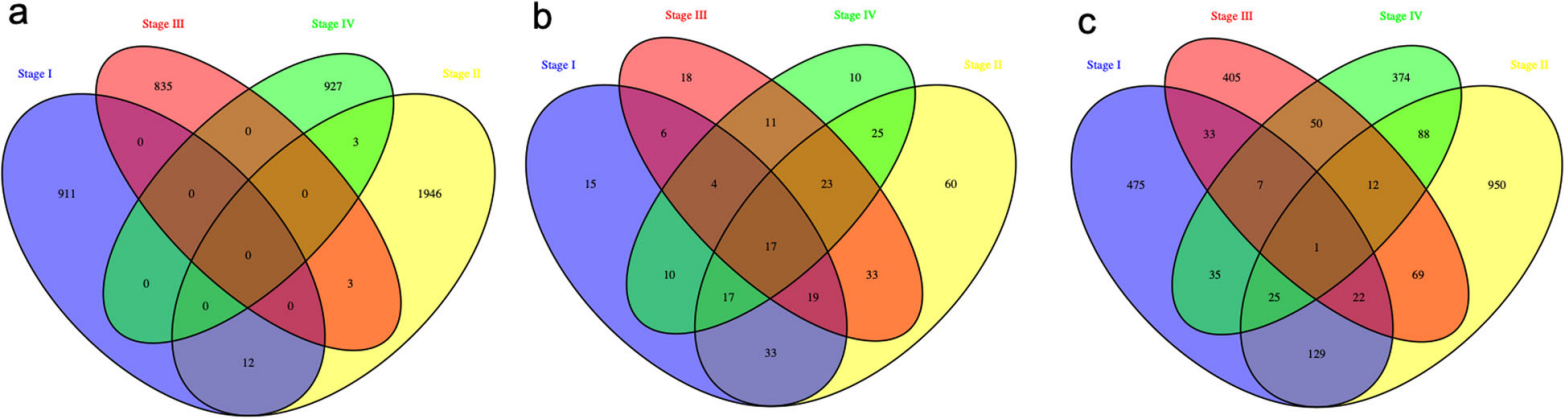
Venn diagrams of miRNA-gene associations identified in each pathological stage. **a** Comparison of miRNA-gene associations among four stages; **b** Comparison of miRNAs associated with gene expression among four stages; **c** Comparison of genes in association with miRNAs among four stages

**Fig. 3:**
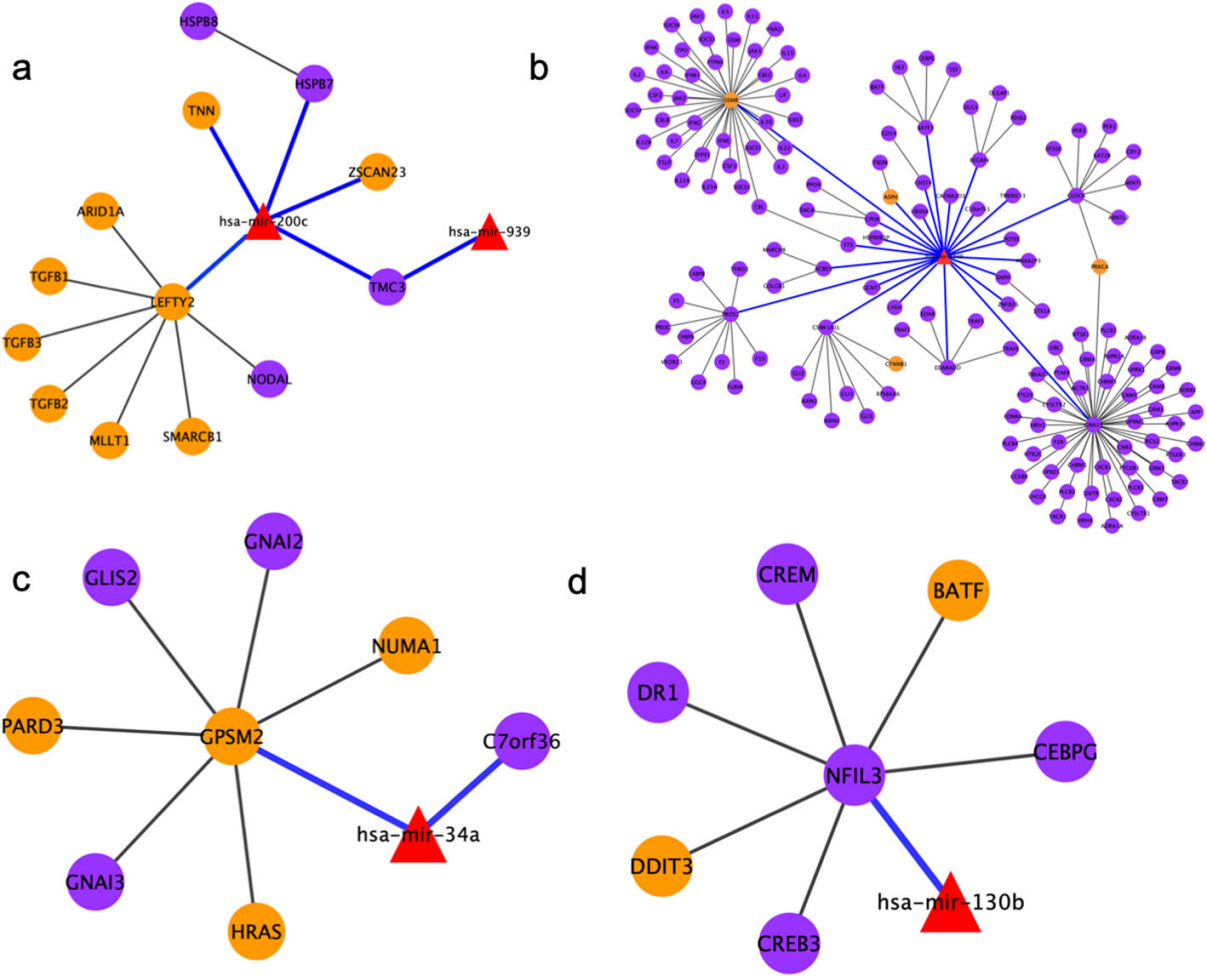
Examples of miRNA-gene subnetworks specific to each stage. Purple nodes represent genes, orange nodes denote cancer genes, and red triangle nodes represent miRNAs that are associated with genes. Gene network expansion are black lines and miRNA-gene associations are solid blue lines. **a** A subnetwork specific to stage I; **b** A subnetwork specific to stage II; **c** A subnetwork specific to stage III; **d** A subnetwork specific to stage IV

From our miRNA and gene expression association analysis, we identified 923, 1964, 838 and 930 significant miRNA-gene associations for stage I, II, III and IV, separately (Table 1). We observed that the distances between miRNAs and their associated genes in different distance windows follow a similar trend but they do differ among different pathological stages. We found that these miRNA-gene associations are stage specific and there is no overlap miRNA-gene pairs among all stages although there 17 miRNAs and one gene shared among these stages (Fig. 2). The numbers of significant associations identified may be confounded by the varied sample sizes in different stages (31 for stage I, 82 for stage II, 59 for stage III and 23 for stage IV). However, this observation that different stages share little in miRNA-gene associations, implies that miRNAs may affect gene expression differently in varied pathological stages and this may indicate disease progression and etiology. We admit that this little sharing of miRNA-gene pairs in different stages might also be partially due to the limited power of association analysis resulted from small sample sizes. Nonetheless, some of the stage-specific miRNAs have been previously reported to affect the progression of colon cancer. For example, *hsa*−*mir*−200*c* was reported to be related to colon cancer progression [42] that was found to be associated with cancer genes in stage I but not in other stages in our results (Fig. 3a). *hsa*−*mir*−1249 which was shown to up-regulate genes in colon cancer [43] and was found to be associated with *OSMR* that affect the progression of colon cancer in stage II in our association study [44] (Fig. 3b). *hsa*−*mir*−34*a* was reported to up-regulate or down-regulate target genes in colon cancer in stage III [45] (Fig. 3c). In stage IV, *hsa*−*mir*−130*b* was reported to affect the tumor progress in colon cancer. [14, 46, 47] which was associated with cancer gene *GPSM*2 in our study [48] (Fig. 3d). In the miRNA-gene associations, we also found some stage-specific genes were reported to be associated with colon cancer in literature. For example, *PLA*2*G*2*A* was only identified to be associated with *hsa*−*mir*−129−1 in stage I, and was previously reported to have low expression in colon cancer patients [49] and *hsa*−*mir*−129−1 was reported to act as a tumor suppressor [50]. Another gene *MCC* was only found to be associated with *hsa*−*mir*−671 in stage II that was reported to have somatic mutations in colon cancer [51]. Moreover, the associated miRNA *hsa*−*mir*−671 was reported to up-regulate the gene expression in colon cancer study [52]. Another gene associated with colon cancer was *FGFR*3 [53], and the deregulated expression of the associated miRNA *hsa*−*mir*−9, only identified in Stage IV, had an important role in colon cancer progression [54]. All these examples demonstrated that some of these miRNAs and genes specifically associated in different pathological stages are reported to be related to colon cancer, and can thus serve as potential biomarkers and anchor points toward further understanding of the progression and prognosis of colon cancer.

**Table 1:** The distance distribution between miRNAs and their associated genes

Stage	(0,1MB]	(1,10MB]	(10,50MB]	(50,10MB]	(100,200MB]	CrossChr
I	2	5	9	9	1	897
II	1	5	25	20	13	1900
III	3	17	21	11	7	779
IV	1	4	11	3	6	905

### Gene Ontology enrichment analysis

**Fig. 4:**
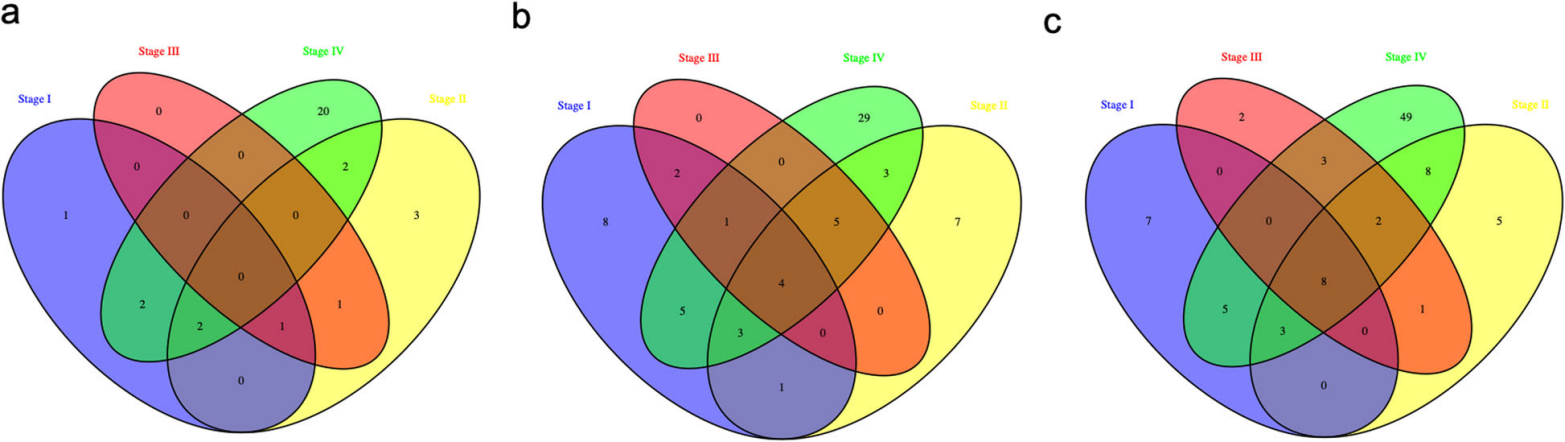
GO enrichment analysis of gene associated with miRNAs in four stages. a. Enriched molecular functions; b. Enriched cellular components; c. Enriched biological processes

We conducted GO enrichment analysis for the miRNA associated genes and found that 6 molecular functions, 23 biological processes, 24 cellular components were enriched for stage I; 9 molecular functions, 30 biological processes, 23 cellular components were enriched, and 5 biological process depleted for stage II; 2 molecular functions, 16 biological processes, 12 cellular components were enriched, and 2 biological process depleted for stage III; and 26 molecular functions, 78 biological processes, 50 cellular components were enriched for stage IV (Additional file 1). The comparison of molecular function, biological process and cellular component are showed in Fig. 4. Besides the common enriched cellular components, molecular functions and biological processes, there were different functions in these four categories enriched by target and associated genes through our GO analysis. For example, biological processes that were related to colon cancer such as chemotaxis and regulation of cell projection organization, were particularly enriched in stage I; cellular process and cell adhesion were particularly enriched in stage II; gene expression were particularly enriched in stage III and cell differentiation and regulation of immune system process were particularly enriched in stage IV [55]. Molecular functions that were related to colon cancer such as sodium ion transmembrane transporter activity were particularly enriched in stage I; growth factor activity and receptor regulator activity was particularly enriched in stage II; and chemokine activity and ion channel activity was particularly enriched in stage IV [55]. Cellular components that were related to colon cancer such as exocytic vesicle and synaptic vesicle were particularly enriched in stage I; cytoplasmic part and cytoplasm was particularly enriched in stage II; membrane protein complex and integral component of lumenal side of endoplasmic reticulum membrane was particularly enriched in stage IV [55]. It is thus important to investigate the particularly enriched biological processes, molecular functions, and cellular components that are specific to certain pathological stage. Such investigations will help shed light on how miRNAs play a different role in regulating gene expression in various pathological stages and thus can serve as biomarkers or signature profiles to help screen, subtype, diagnose and treat patients.

### Network analysis and alignment

First, we checked how the genes are interacting with each other in the genes targeted by or associated with miRNAs in each pathological stage (Additional file 2). We found that 98.87% (12420/12562) of these genes are interacting according to the protein protein interactions with high confidence in the InWeb database where we set the confidence score cutoff at 0.2 (Table 2).

**Table 2:** The numbers of genes targeted by and associated with miRNAs, and their interactions in each pathological stage

Stage	Number of targeted and associated genes	Number of interactions
I	10358	39002
II	13822	54858
III	11273	44490
IV	11613	42122

**Fig. 5:**
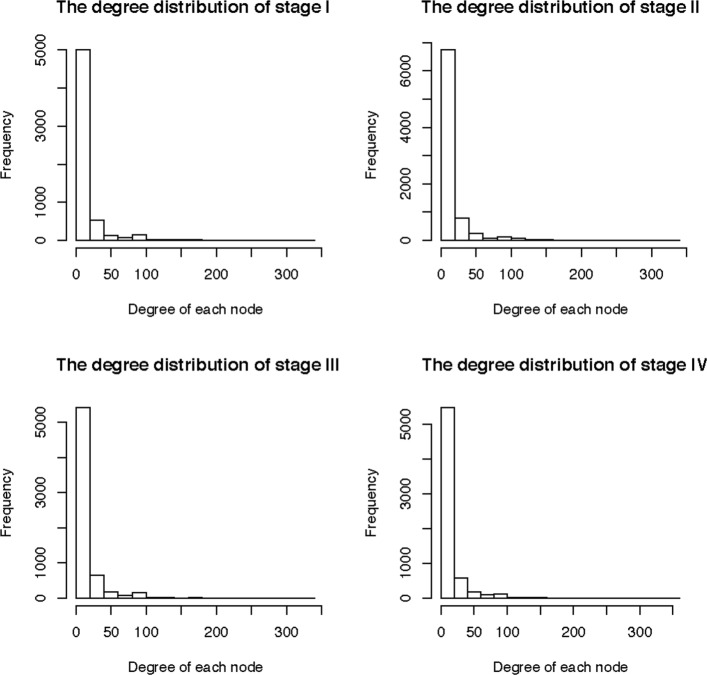
The histogram of degree distribution in the miRNA-gene network of each stage

Second, we compared the four integrated miRNA-gene networks of different stages by comparing important network properties of these networks such as the edge similarity, centrality of vertices, diameter of graph, and betweenness of the network in each pathological stage (Table 3). The edge similarity was calculated as the proportion of common edges of the network for stage I and all other networks for the remaining three stages. The centrality of vertices denoted the importance of the vertices in the network [56] with higher values implying that the nodes were closer to the center of the network. The betweenness of vertices denoted the number of the shortest paths passing through vertices [57]. The higher the betweenness of a node, the more of other nodes can reach the center by the shortest path through this node. We use the average of centrality and betweenness of all nodes in the network in Table 3. Another measurement we used was the diameter of each network of each stage which measured the longest path from one vertex to another in the network. We also investigated the degree distribution of vertices of network in each stage (Fig. 5). These network properties showed that there was different pattern between stage I and other stages, which provided evidence on different effect of miRNAs on gene expression in different pathological stages. Particularly, we observed that pathological stage II is the most different comparing with networks of the other stages, and stage III and IV are more similar. The network of stage II is most similar to stage I, has the largest diameter, has the lowest centrality where nodes are less important comparing to other networks, and has the largest betweenness with more nodes reaching to network centers via shortest paths. Hence, these results suggest that we need to investigate why stage II has distinct network property comparing to other stages to better understand disease progression and prognosis.

**Table 3:** Comparison of network properties of the integrated miRNA-gene networks in four stages

Properties	I	II	III	IV
Components	33	30	27	28
Edge similarity	NA	0.8826	0.7965	0.7338
Diameter	5986	8108	6487	6449
Centrality	2.23E-06	1.72E-06	2.31E-06	2.22E-06
Betweenness	8959.615	12861.60	9878.94	9834.99
Edge density	0.002278	0.001721	0.002148	0.002076

Finally, we conducted network alignment to find local network structures that differ in the integrated miRNA-gene networks in the four pathological stages. We found many examples that miRNAs affect gene expression with different patterns among varied pathological stages (Fig. 3). For example, Fig. 3a showed one miRNA *hsa*−*mir*−200*c* was related to colon cancer progression [42] that was associated with some cancer genes only in stage I. *hsa*−*mir*−1249 which was reported to up-regulate genes in colon cancer [43] was associated with *OSMR* in stage II and *OSMR* affects the progression of colon cancer [44]. In addition, another gene *CTNNB*1 reported to affect colon cancer progression was also involved in this miRNA-gene network in stage II [58]. *hsa*−*mir*−34*a* was reported to up-regulate or down-regulate target genes in colon cancer and was found to be associated with genes changes only in stage IV [45]. In stage IV, *hsa*−*mir*−130*b* was reported to affect the tumor progression in colon cancer. [14, 46, 47] which is associated with cancer gene *GPSM*2 in our study [48]. These four networks are only present in each corresponding stage and do not overlap cross stages which provide the evidence of our assumption that the miRNA-gene network has different patterns in colon cancer pathological stages.

## Discussion and conclusion

This study implemented an association and network integration strategy to help understand the underlying mechanism on how miRNAs distinctively affect gene expression in different pathological stages of colon cancer. Our results showed that there is significant difference among the miRNA-gene associations and interactions in different pathological stages. Particularly, pathological stage II was notably different from other stages regarding to their network properties in our miRNA-gene network analysis. We believe that such analysis will point to potential follow-up studies dedicated to investigating molecular mechanisms that lead to these differences. With more samples with clinical information available, together with matching genetic and epigenetic profiles, we expect to apply the strategy designed in this study to find more detailed delineations of regulatory changes in different pathological stages and diseases. It is also worth noting that this method is applicable to any cancer type or even other disease that has clinical profile data available.

In summary, we found that there is significant statistical difference on the miRNA-gene interactions between different pathological stages in colon cancer, which supported by other studies that network signature plays different roles in the pathogenesis [59, 60], metastasis [61] and the tumorigenesis of colon cancer. Our approach for integrating different types of data (including but not limited to miRNA expression, gene expression, protein protein interactions) can reveal the interactions and cross-talks between multi-layers of genetic components in interpreting the etiology, prognosis and progression of various diseases including different cancer types. Given the evidence that these interactions and network communities differ in different disease stages, this study also shed light on potential mitigation or treatment plan that helps deter or redirect the progression of diseases through diverting or changing the network structure in a systems biology way.

## Data Availability

The data, code, and results of this study can be publicly accessed via github at https://github.com/shilab/MicroRNA-Gene-Network-Colon/.

## Abbreviations

EMT: pithelial-mesenchymal transition
GO: Gene Ontology
TCGA: The Cancer Genome Atlas
